# Analysis of dispersion and absorption characteristics of shear waves in sinusoidally corrugated elastic medium with void pores

**DOI:** 10.1098/rsos.160511

**Published:** 2017-02-01

**Authors:** Deepak Kr. Pandit, Santimoy Kundu, Shishir Gupta

**Affiliations:** Department of Applied Mathematics, Indian Institute of Technology (Indian School of Mines), Dhanbad, Jharkhand 826004, India

**Keywords:** shear waves, corrugation, void pores, sandiness, dispersion, absorption

## Abstract

This theoretical work reports the dispersion and absorption characteristics of horizontally polarized shear wave (SH-wave) in a corrugated medium with void pores sandwiched between two dissimilar half-spaces. The dispersion and absorption equations have been derived in a closed form using the method of separation of variables. It has been established that there are two different kinds of wavefronts propagating in the proposed media. One of the wavefronts depends on the modulus of rigidity of elastic matrix of the medium and satisfies the dispersion equation of SH-waves. The second wavefront depends on the changes in volume fraction of the pores. Numerical computation of the obtained relations has been performed and the results are depicted graphically. The influence of corrugation, sandiness on the phase velocity and the damped velocity of SH-wave has been studied extensively.

## 1. Introduction

In the last few decades, the studies on propagation of shear wave (SH-wave) through isotropic media have been of outstanding attention, as it served to analyse the Earth's interior structure. The seismological research has focused on specific areas of the Earth, such as the crust, mantle, outer/inner core and the interfaces between different strata. It is our great interest to examine the propagation of SH-waves in an elastic isotropic medium with void pores as it plays an indispensable function in material fracture and failure. Originally, Cowin & Nunziato [1] developed a nonlinear theory of elastic solids with voids. Later, they [2] introduced the linear theory of elastic materials as a specialization of the article [1] which is a valuable extension to the classical theory of elasticity. The study interpreted that the linear theory differs quite considerably from the classical linear elasticity as the volume fraction was considered as an independent kinematical variable.

Some remarkable works on the propagation of SH-waves in various media with different geometries have been studied by Dey *et al.* [3–5]. Singh [6] studied the reflection and transmission of couple longitudinal waves at a plane interface between two dissimilar half-spaces of thermo-elastic materials with voids. Khan *et al.* [7] observed the gravitational effect on surface waves in homogeneous fibre-reinforced anisotropic general viscoelastic media of higher and fractional order with voids. Rani *et al.* [8] studied the torsional vibrations of initially stressed composite poroelastic cylinders. Golamhossen [9] studied the propagation of waves in an elastic cylinder with voids. Vishwakarma *et al.* [10] discussed the influence of the rigid boundary on the Love wave propagation in an elastic layer with void pores.

It has been observed by many articles that the materials of various layers under the surface of the Earth are not always elastic or isotropic. It may be a sandy medium consisting of sandy particles having no moisture or water vapour. The resistance to shear is much less in dry sandy medium because of individual granules' slippage over each other; for that reason the granules of sandy medium are very much responsible for greater shear deflection [11]. According to the classical theory of elasticity, Young's modulus, modulus of rigidity and Poisson's ratio are related by the expression $E/\mu_{2}=2(1+\nu )$, where *E* is Young's modulus, $\mu_{2}$ is the rigidity of elastic medium and *ν* is Poisson's ratio. The relation may be rewritten for dry sandy medium as

$$\frac{E}{\mu_{2}}=2\eta (1+\nu ),$$

where *η* is the sandiness parameter. The sandiness parameter η>1 corresponds to the sandy medium and η=1 for an elastic medium. The value of Young's modulus *E* when compared with modulus of rigidity $\mu_{2}$ is much greater in sandy medium than a solid material. Recently, some research articles on the propagation of surface wave in dry sandy medium have been studied by Kundu *et al.* [12], Pal & Ghorai [13] and Dey *et al.* [14].

Generally, the interfaces between two layers of the Earth's interior structure can neither be perfectly flat nor in a regular shape to a certain extent. However, some boundaries/interfaces may be approximated as regular ones, while others may be considered as irregular. The effects of irregular boundary surfaces on the wave propagation have been studied in many previous articles, such as Zhang & Shinozuka [15], Singh [16], Chattopadhyay & Singh [17] and many more. The shape of irregular interfaces may be in different forms, *viz*. rectangular, triangular, parabolic, corrugated, etc. The propagation and attenuation of waves through such type of corrugated boundaries get affected by these undulations or roughness. The reflection and transmission of SH-waves at a corrugated interface between two laterally and vertically heterogeneous anisotropic elastic solid half-spaces has been investigated by Tomar & Kaur [18]. Later, they [19] have shown the reflection and refraction of SH-waves at a corrugated interface between two laterally and vertically heterogeneous viscoelastic solid half-spaces. Recently, Kundu *et al.* [20] have shown the effects of periodic corrugation, reinforcement, heterogeneity and initial stress on the propagation of Love waves. Singh *et al.* [21] studied the influence of corrugated boundary surfaces, reinforcement, hydrostatic stress, heterogeneity and anisotropy on Love-type wave propagation. Chattaraj & Samal [22] obtained the dispersion of Love-type surface wave in anisotropic porous layer with periodic non-uniform boundary surface.

To the best of the authors' knowledge, there are no analytical results available in the literature for the SH-wave propagation in a sinusoidally corrugated elastic medium with void pores lying between two dissimilar half-spaces. Consequently, this motivates us to examine the influence of corrugated boundary surfaces and sandiness parameter on the propagation of SH-waves in the proposed media.

The objective of this issue is to characterize the influence of corrugated boundary surfaces and sandiness on the propagation of SH-waves in the corrugated elastic medium with void pores sandwiched between two half-spaces. It has been considered that elastic medium with void pores is bounded by two sinusoidally corrugated surfaces $z=-H+f_{1}(x)$ and $z=f_{2}(x)$. $\;\;f_{1}(x)$ and $f_{2}(x)$ are the continuous periodic functions of *x*, independent of *y* (see §2). The dispersion and absorption relations are introduced in closed simple form. It is shown that there is a possibility of propagation of SH-waves in two different wavefronts, one of the wavefronts is associated with the elasticity of the medium and the other wavefront is associated with the changes in void's volume fraction.

## 2. Formulation of physical model and its solution

**Figure 1.**
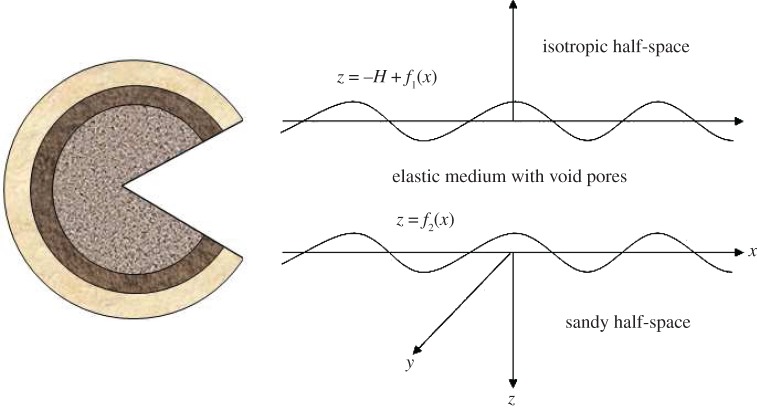
Structure of the problem.

We consider a corrugated elastic medium with void pores sandwiched between an isotropic half-space and a sandy half-space as shown in figure 1. A rectangular Cartesian coordinate system (x,y,z) is taken in such a way that *x*-axis is taken along the direction of wave propagation and *z*-axis is pointing vertically downward into the lower half-space. Since the constituent particles of the media are aligned on a line parallel to *y*-axis and are displacing equally, the field quantities are independent of *y*-direction, i.e. ∂/∂y≡0. The average thickness of the corrugated layer is assumed to be *H*. The average thickness of the corrugated layer may be defined as $f_{1}(x)-H\leq z\leq f_{2}(x)$; and the upper and lower half-spaces as $-\infty \leq z\leq f_{1}(x)-H$ and $f_{2}(x)\leq z\leq \infty ,$ respectively, where the Fourier series expansion of the functions $f_{1}(x)$ and $f_{2}(x)$ may be given as [16]

2.1 $$f_{j}(x)=\sum_{n=1}^{\infty }(\zeta_{n}^{j}\;e^{inbx}+\zeta_{-n}^{j}\;e^{-inbx});\;j=1,2\ldots ,$$

where $\zeta_{n}^{j}$ and $\zeta_{-n}^{j}$ are the Fourier series expansion coefficients, 2π/b is the corrugation wavelength, *n* is the series expansion order and i is $\sqrt{-1}$. Now let us define the constants $a_{j}$, $a_{n}^{j}$ and $b_{n}^{j}$ in such a way that $\zeta_{\pm 1}^{j}=a_{j}/2$ and $\zeta_{\pm n}^{j}=(a_{n}^{j}\mp ib_{n}^{j})/2$; (n=2,3…). Therefore, the Fourier series (2.1) may be expressed as

2.2 $$f_{j}(x)=a_{j}\cos(bx)+\sum_{n=2}^{\infty }(a_{n}^{j}\cos(nbx)+b_{n}^{j}\sin(nbx));\;j=1,2,$$

In this article, we shall consider the above series expansion is up to first order, hence the boundary surfaces may be expressed as only one cosine term, i.e. $f_{i}(x)=a_{i}\cos(bx)$, i=1,2, where $a_{i}$ are the corresponding amplitudes of the undulated surface. It will be convenient to describe the undulated nature on the propagation and attenuation of shear waves.

*Shear-wave condition*. Let the displacement components of upper isotropic half-space, elastic medium with void pores and lower half-space be $\left(u_{0},v_{0},w_{0}\right)$, $\left(u_{1},v_{1},w_{1}\right)$ and $\left(u_{2},v_{2},w_{2}\right)$, respectively, so that the condition for the propagation of SH-waves may be given by

2.3 $$u_{i}(x,z,t)=0=w_{i}(x,z,t)\;\mathrm{and}\;v_{i}=v_{i}(x,z,t);\;i=1,2,3.$$

### 2.1. Wave propagation in the isotropic half-space

The equations of motion for the propagation of SH-wave in an isotropic elastic solid in the absence of body forces may be written in compound form as Biot [23]

2.4 $$\sigma_{ij,j}=\rho_{0}{\ddot{u}}_{i};\;i,j=1,2,3,$$

where $\sigma_{ij}$ are the stress components and $\rho_{0}$ is the density of the medium. Equation (2.4) may be written as

2.5 $$\begin{array}{rl} & \frac{\partial \sigma_{11}}{\partial x}+\frac{\partial \sigma_{12}}{\partial y}+\frac{\partial \sigma_{13}}{\partial z}=\rho_{0}\frac{\partial^{2}u_{0}}{\partial t^{2}};\;\frac{\partial \sigma_{21}}{\partial x}+\frac{\partial \sigma_{22}}{\partial y}+\frac{\partial \sigma_{23}}{\partial z}=\rho_{0}\frac{\partial^{2}v_{0}}{\partial t^{2}}\\ \text{and}\;\; & \frac{\partial \sigma_{31}}{\partial x}+\frac{\partial \sigma_{32}}{\partial y}+\frac{\partial \sigma_{33}}{\partial z}=\rho_{0}\frac{\partial^{2}w_{0}}{\partial t^{2}}. \end{array}\}$$

Applying Hooke's Law for an isotropic medium gives

2.6 $$\sigma_{ij}=\lambda \Delta \delta_{ij}+2\mu_{0}e_{ij},$$

where *λ*, Δ, *δ*_*ij*_, $\mu_{0}$ are Lame's constant, cubical dilation, Kronecker delta function and rigidity of the medium, respectively, and $e_{ij}=\frac{1}{2}(\partial u_{i}/\partial x_{j}+\partial u_{j}/\partial x_{i})$ are components of strain tensor.

Now, using the SH-wave condition, the equation of motion get reduced to

2.7 $$\frac{\partial^{2}v_{0}}{\partial x^{2}}+\frac{\partial^{2}v_{0}}{\partial z^{2}}=\frac{1}{\beta_{0}^{2}}\frac{\partial^{2}v_{0}}{\partial t^{2}},$$

where $\beta_{0}=\sqrt{\mu_{0}/\rho_{0}}$ is the SH-wave velocity in the isotropic medium.

Considering the solution of (2.7) as

2.8 $$v_{0}=V_{0}(z)\;e^{ik(x-ct)},$$

such that $V_{0}(z)$ satisfies the equation

2.9 $$V_{0}^{\prime \prime }(z)-s_{0}^{2}V_{0}(z)=0,$$

where $s_{0}=k\sqrt{1-c^{2}/\beta_{0}^{2}}$. The appropriate expression for the displacement component in the upper isotropic half-space may be obtained as

2.10 $$V_{0}(z)=A_{0}e^{s_{0}z}+A_{1}e^{-s_{0}z}.$$

Therefore, in view of equation (2.8), we get

2.11 $$v_{0}(x,z,t)=(A_{0}e^{s_{0}z}+A_{1}e^{-s_{0}z})\;e^{ik(x-ct)},$$

where *k* is wavenumber, *c* is wave velocity and *t* is time.

The solution of upper half-space vanishes as z→−∞. Hence, the following equation:

2.12 $$v_{0}(x,z,t)=A_{0}e^{s_{0}z}\;e^{ik(x-ct)}$$

is the displacement component of the isotropic upper half-space.

### 2.2. Wave propagation in elastic medium with void pores

The field equations governing the displacement u(x,t) and the volume fraction ϕ(x,t) for a homogeneous and isotropic elastic medium in the absence of body forces as given by Cowin & Nuziato [1] are

2.13 $$\mu \nabla^{2}u+(\lambda +\mu )\nabla (\nabla .u)+\gamma \nabla \phi =\rho \frac{\partial^{2}u}{\partial t^{2}}$$

and

2.14 $$\alpha \nabla^{2}\phi -\xi \phi -\omega \frac{\partial \phi }{\partial t}-\gamma \nabla .u=\rho \bar{k}\frac{\partial^{2}\phi }{\partial t^{2}},$$

α,γ,ξ,ω are the parameters of material due to void field; $\bar{k}$ is equilibrated inertia, *ρ* is the density of the medium.

The above equations of motion are welded by the following constitutive equations:

2.15 $$\tau_{ij}=\lambda \delta_{ij}e_{kk}+2\mu e_{ij}+\gamma \phi \delta_{ij};\;i,j=1,2,3,$$

where $\tau_{ij}$ are components of stress tensor. Moreover,

2.16 $$\mu \geq 0,\;\alpha \geq 0,\;\xi \geq 0,\;\bar{k}\geq 0,\;\bar{k}\xi \geq \gamma^{2}\;\mathrm{and}\;\omega \geq 0,\;\mathrm{where}\;\bar{k}=\lambda +(\frac{2}{3})\mu .$$

Again using the SH-wave condition, the non-vanishing equations of motion (2.13) and (2.14) in elastic medium with void pores in the absence of body forces may be written as

2.17 $$\mu \left(\frac{\partial^{2}v_{1}}{\partial x^{2}}+\frac{\partial^{2}v_{1}}{\partial z^{2}}\right)+\gamma \left(\frac{\partial \phi }{\partial x}+\frac{\partial \phi }{\partial z}\right)=\rho \frac{\partial^{2}v_{1}}{\partial t^{2}}$$

and

2.18 $$\alpha \left(\frac{\partial^{2}\phi }{\partial x^{2}}+\frac{\partial^{2}\phi }{\partial z^{2}}\right)-\xi \phi -\omega \frac{\partial \phi }{\partial t}=\rho \bar{k}\frac{\partial^{2}\phi }{\partial t^{2}}.$$

Let us consider the solution of equations (2.17) and (2.18) as

2.19 $$v_{1}=\bar{v_{1}}(z)\;e^{ik(x-ct)}\;\mathrm{and}\;\phi =\bar{\phi }(z)\;e^{ik(x-ct)},$$

where $\bar{v_{1}}(z)$ and $\bar{\phi }(z)$ satisfy the equations

2.20 $${\bar{v_{1}}}^{\prime \prime }(z)-N^{2}\bar{v_{1}}(z)+\frac{\gamma }{\mu }[ik\bar{\phi }(z)+{\bar{\phi }}^{\prime }(z)]=0$$

and

2.21 $${\bar{\phi }}^{\prime \prime }(z)-M^{2}\bar{\phi }(z)=0,$$

respectively, where $N=k{\left(1-c^{2}/\beta^{2}\right)}^{1/2}$; $M={\left(k^{2}-\rho \bar{k}k^{2}c^{2}/\alpha -i\omega kct/\alpha +\xi /\alpha \right)}^{1/2}$ and $\beta =\sqrt{\mu /\rho }$ is SH-wave velocity in elastic void pores. Therefore, the solution of equations (2.17) and (2.18) may be expressed as

2.22a $$v_{1}(x,z,t)=(B_{1}e^{Nz}+B_{2}e^{-Nz}+B_{3}X_{1}e^{Mz}+B_{4}X_{2}e^{-Mz})\;e^{ik(x-ct)}$$

and

2.22b $$\phi (x,z,t)=(B_{3}e^{Mz}+B_{4}e^{-Mz})e^{ik(x-ct)},$$

where $X_{1}=-\gamma (ik+M)/\mu (M^{2}-N^{2})$ and $X_{2}=-\gamma (ik-M)/\mu (M^{2}-N^{2})$.

### 2.3. Wave propagation in dry sandy half-space

With the help of equation (2.3) and using the stress–strain relations [23], the components of stress for the concerned medium may be procured as follows:

2.23 $$S_{11}=0,\;S_{22}=0,\;S_{33}=0,\;S_{12}=S_{21}=\frac{\mu_{2}}{\eta }\frac{\partial v_{2}}{\partial x},\;S_{13}=S_{31}=0\;\mathrm{and}\;S_{23}=S_{32}=\frac{\mu_{2}}{\eta }\frac{\partial v_{2}}{\partial z}.$$

Therefore, the non-vanishing equation of motion for the propagation of SH-waves in the dry sandy medium with rigidity $\mu_{2}/\eta$ and density $\rho_{2}$ is given by

2.24 $$\frac{\mu_{2}}{\eta }\left(\frac{\partial^{2}v_{2}}{\partial x^{2}}+\frac{\partial^{2}v_{2}}{\partial z^{2}}\right)=\rho_{2}\frac{\partial^{2}v_{2}}{\partial t^{2}}$$

considering the solution of equation (2.24) as

2.25 $$v_{2}(x,z,t)=V_{2}(z)\;e^{ik(x-ct)},$$

such that $V_{2}(z)$ satisfy the equation

2.26 $$V_{2}^{\prime \prime }(z)-s_{2}^{2}V_{2}(z)=0,$$

where $s_{2}=k\sqrt{1-c^{2}/\beta_{2}^{2}}$ and $\beta_{2}(=\sqrt{\mu_{2}/\eta \rho_{2}})$ is SH-wave velocity of the sandy medium. The solution of equation (2.26) may be obtained as

2.27 $$V_{2}(z)=A_{2}e^{-s_{2}z}+A_{3}e^{s_{2}z},$$

which gives

2.28 $$v_{2}(x,z,t)=(A_{2}e^{-s_{2}z}+A_{3}e^{s_{2}z})\;e^{ik(x-ct)}.$$

Since, the displacement component vanishes as z→∞, the expression for the displacement component in the lower half-space as

2.29 $$v_{2}(x,z,t)=A_{2}e^{-s_{2}z}\;e^{ik(x-ct)}.$$

## 3. Boundary conditions and frequency equations

The required boundary conditions for the concerned problem include the continuity of the stress tensors, displacements and volume fraction at the solid interfaces. Therefore, at the corrugated interfaces some suitable boundary conditions may be mathematically given as follows:

Mathematically,

3.1 $$\begin{array}{rl} 1.\;\mathrm{At}\;z=f_{1}(x)-H,\; & \left(i\right)\;v_{0}=v_{1}, \end{array}$$

3.2 $$\begin{array}{rl} & \left(\mathrm{ii}\right)\;\sigma_{23}-f_{1}^{\prime }\sigma_{12}=\tau_{23}-f_{1}^{\prime }\tau_{12} \end{array}$$

3.3 $$\begin{array}{rl} & \left(\mathrm{iii}\right)\;\bar{n}.\nabla \phi =0 \end{array}$$

3.4 $$\begin{array}{rl} 2.\;\mathrm{At}\;z=f_{2}(x),\; & \left(i\right)\;v_{1}=v_{2} \end{array}$$

3.5 $$\begin{array}{rl} & \left(\mathrm{ii}\right)\;\tau_{23}-f_{2}^{\prime }\tau_{12}=S_{23}-f_{2}^{\prime }S_{12} \end{array}$$

3.6 $$\begin{array}{rl} & \left(\mathrm{iii}\right)\;\bar{n}.\nabla \phi =0, \end{array}$$

where $\bar{n}$ is the unit vector normal to the external boundary and $f_{i}^{\prime }=\partial f_{i}^{\prime }(x)/\partial x$; *i* = 1,2.

Substituting equations (2.12), (2.22*a*,*b*) and (2.29) on the above boundary conditions (3.1)–(3.6), following equations may be obtained:

3.7 $$\begin{array}{rl} & e^{(\;f_{1}-H)s_{0}}A_{0}-e^{N(\;f_{1}-H)}B_{1}-e^{-N(\;f_{1}-H)}B_{2}-e^{M(\;f_{1}-H)}B_{3}X_{1}-e^{-M(\;f_{1}-H)}B_{4}X_{2}=0 \end{array}$$

3.8 $$\begin{array}{rl} & e^{(\;f_{1}-H)s_{0}}A_{0}(-if_{1}^{\prime }k+s_{0})\frac{\mu_{0}}{\mu_{1}}+e^{N(\;f_{1}-H)}(if_{1}^{\prime }k-N)B_{1}+ie^{-N(\;f_{1}-H)}(f_{1}^{\prime }k-iN)B_{2}\\ & \;+e^{M(\;f_{1}-H)}(if_{1}^{\prime }k-M)B_{3}X_{1}+e^{-M(\;f_{1}-H)}(if_{1}^{\prime }k+M)B_{4}X_{2}=0 \end{array}$$

3.9 $$\begin{array}{rl} & e^{M(\;f_{1}-H)}MB_{3}-e^{-M(\;f_{1}-H)}MB_{4}=0 \end{array}$$

3.10 $$\begin{array}{rl} & \;-e^{f_{2}s_{0}}A_{2}+e^{Nf_{2}}B_{1}+e^{-Nf_{2}}B_{2}+e^{Mf_{2}}B_{3}X_{1}+e^{-Mf_{2}}B_{4}X_{2}=0 \end{array}$$

3.11 $$\begin{array}{rl} & e^{-f_{2}s_{2}}A_{2}(if_{2}^{\prime }k+s_{2})\frac{\mu_{2}}{\eta \mu_{1}}+e^{Nf_{2}}(-if_{2}^{\prime }k+N)B_{1}-ie^{-Nf_{2}}(f_{2}^{\prime }k-iN)B_{2}\\ & \;+e^{Mf_{2}}(-if_{2}^{\prime }k+M)B_{3}X_{1}+e^{-Mf_{2}}(-if_{2}^{\prime }k-M)B_{4}X_{2}=0 \end{array}$$

3.12 $$\begin{array}{rl} \text{and}\;\; & e^{Mf_{2}}MB_{3}-e^{-Mf_{2}}MB_{4}=0 \end{array}$$

Eliminating the arbitrary constants $A_{0},B_{1},B_{2},B_{3},B_{4},A_{2}$ from above equations (3.7)–(3.12) yields

3.13 $$\left(\tan[\Gamma_{1}+i\Gamma_{2}]-\frac{T_{1}+iT_{2}}{T_{3}+iT_{4}}\right)M^{2}=0,$$

where $\Gamma_{1}$, $\Gamma_{2}$, $T_{1}$, $T_{2}$, $T_{3}$ and $T_{4}$ are provided in appendix A.

Equation (3.13) implies that

3.14 $$\tan[\Gamma_{1}+i\Gamma_{2}]=\frac{T_{1}+iT_{2}}{T_{3}+iT_{4}}$$

or

3.15 $$M^{2}=0.$$

The above equations elucidate that the SH-wave propagates through elastic medium with void pores in two wavefronts. Equations (3.14) and (3.15) are the required frequency equations of SH-wave of the first and second kind, respectively, in elastic medium with void pores between two dissimilar half-spaces. It is also noticed that these frequency equations are in complex-valued form and are implicit equations. Owing to dissipation of the elastic solids, waves are attenuated. If the dissipation coefficient is non-zero, the wavenumber *k* should be complex, i.e. $k=k_{1}+ik_{2}$. Therefore, separating the real and imaginary parts of equation (3.14) yields

3.16 $$\frac{\sin(2\Gamma_{1})}{\cos(2\Gamma_{1})+\cosh(2\Gamma_{2})}=\frac{T_{1}T_{3}+T_{2}T_{4}}{{T_{3}}^{2}+{T_{4}}^{2}}$$

and

3.17 $$\frac{\sinh(2\Gamma_{2})}{\cos(2\Gamma_{1})+\cosh(2\Gamma_{2})}=\frac{T_{2}T_{3}-T_{1}T_{4}}{{T_{3}}^{2}+{T_{4}}^{2}},$$

respectively. With the similar approach, the separation of real and imaginary part of equation (3.15) gives

3.18 $$(\delta^{2}-1)\left(\frac{c_{2}^{2}}{c_{v}^{2}}-1\right)+\frac{1}{{\left(k_{1}R\right)}^{2}}+\frac{c_{2}\delta \kappa t}{c_{v}{\left(k_{1}R\right)}^{2}}=0$$

and

3.19 $$2\delta \left(1-\frac{c_{2}^{2}}{c_{v}^{2}}\right)-\frac{c_{2}\kappa t}{c_{v}{\left(k_{1}R\right)}^{2}}=0,$$

respectively, where $\delta (=k_{2}/k_{1})$ is attenuation co-efficient, $k_{1}$=Re[k], $\kappa (=\omega c_{v}k_{1}/\xi )$ is a dimensionless parameter, $c_{v}(=\sqrt{\alpha /\rho \bar{k}})$ is the velocity of wave due to change in volume fraction, $R(=\sqrt{\alpha /\xi })$ is the displacement parameter, and $c_{2}$ is the velocity of SH-wave of the second kind.

Equations (3.16) and (3.17) are the dispersion and absorption relations of the first kind of SH-waves, whereas equations (3.18) and (3.19) are the dispersion and absorption relations of the second kind of SH-waves. The dispersion relations corresponds to the dispersion curves and the absorption relation corresponds to the attenuation curves. It is clear that the first kind of SH-waves is associated with the parameters of both elastic half-spaces, without void pores. But the second kind of SH-waves depends only with the parameters of void pores.

## 4. Particular cases

### 4.1. Case I

When the elastic void pores medium is bounded by plane surface $f_{1}(x)=0$ and corrugated surface $f_{2}(x)=a_{2}\cos(bx)$, equations (3.16) and (3.17) become

4.1 $$\frac{\sin(2\Gamma_{11})}{\cos(2\Gamma_{11})+\cosh(2\Gamma_{21})}=\frac{T_{11}T_{31}+T_{21}T_{41}}{{T_{31}}^{2}+{T_{41}}^{2}}$$

and

4.2 $$\frac{\sinh(2\Gamma_{21})}{\cos(2\Gamma_{11})+\cosh(2\Gamma_{21})}=\frac{T_{21}T_{31}-T_{11}T_{41}}{{T_{31}}^{2}+{T_{41}}^{2}},$$

respectively, where $\Gamma_{11}$, $\Gamma_{21}$, $T_{11}$, $T_{21}$, $T_{31}$ and $T_{41}$ are given in appendix A. Equations (4.1) and (4.2) are the dispersion and absorption equations for the propagation of SH-waves in an elastic medium with void pores bounded by upper planar boundary surface and lower corrugated boundary surface sandwiched between two dissimilar half-spaces.

### 4.2. Case II

When the elastic void pores medium is bounded by plane surface $f_{1}(x)=a_{1}\cos(bx)$ and corrugated surface $f_{2}(x)=0$, equations (3.16) and (3.17) become

4.3 $$\frac{\sin(2\Gamma_{12})}{\cos(2\Gamma_{12})+\cosh(2\Gamma_{22})}=\frac{T_{12}T_{32}+T_{22}T_{42}}{{T_{32}}^{2}+{T_{42}}^{2}}$$

and

4.4 $$\frac{\sinh(2\Gamma_{22})}{\cos(2\Gamma_{12})+\cosh(2\Gamma_{22})}=\frac{T_{22}T_{32}-T_{12}T_{42}}{{T_{32}}^{2}+{T_{42}}^{2}},$$

respectively, where $\Gamma_{12}$, $\Gamma_{22}$, $T_{12}$, $T_{22}$, $T_{32}$ and $T_{42}$ are given in appendix A. Equations (4.3) and (4.4) are the dispersion and absorption equations for the propagation of SH-waves in an elastic medium with void pores bounded by upper corrugated boundary surface and lower planar boundary surface sandwiched between two dissimilar half-spaces.

### 4.3. Case III

When the elastic void pores medium is bounded by plane surface $f_{1}(x)=a_{1}\cos(bx)$ and corrugated surface $f_{2}(x)=a_{2}\cos(bx)$ then equations (3.16) and (3.17) become

4.5 $$\frac{\sin(2\Gamma_{13})}{\cos(2\Gamma_{13})+\cosh(2\Gamma_{23})}=\frac{T_{13}T_{33}+T_{23}T_{43}}{{T_{33}}^{2}+{T_{43}}^{2}}$$

and

4.6 $$\frac{\sinh(2\Gamma_{23})}{\cos(2\Gamma_{13})+\cosh(2\Gamma_{23})}=\frac{T_{23}T_{33}-T_{13}T_{43}}{{T_{33}}^{2}+{T_{43}}^{2}},$$

respectively, where $\Gamma_{13}$, $\Gamma_{23}$, $T_{13}$, $T_{23}$, $T_{33}$ and $T_{43}$ are given in appendix A. Equations (4.5) and (4.6) are the dispersion and absorption equations for the propagation of SH-waves in an elastic medium with void pores bounded by two corrugated boundary surfaces sandwiched between two dissimilar half-spaces.

### 4.4. Case IV

When the amplitude of the corrugated boundary surfaces are ignored ($a_{1}=a_{2}=0$), and sandiness of lower half-space is neglected (η=1) and attenuation is neglected (δ=0), ($T_{2}=0,\;T_{4}=0$) dispersion relation (3.16) becomes

4.7 $$\tan\left[kH\sqrt{\frac{c^{2}}{\beta^{2}}-1}\right]=\frac{\sqrt{c^{2}/\beta^{2}-1}\left((\mu_{0}/\mu_{1})\sqrt{1-c^{2}/\beta_{0}^{2}}+(\mu_{2}/\mu_{1})\sqrt{1-c^{2}/\beta_{2}^{2}}\;\right)}{(c^{2}/\beta^{2}-1)-(\mu_{0}/\mu_{1})(\mu_{2}/\mu_{1})\sqrt{1-c^{2}/\beta_{0}^{2}}\sqrt{1-c^{2}/\beta_{2}^{2}}},$$

and the absorption relation (3.17) vanishes identically. Equation (4.7) coincides with the classical equation [24] of SH-waves sandwiched between two dissimilar isotropic half-spaces. The range of existence of the real root of equation (4.7) is $\beta <c<\beta_{0}$ or $\beta_{2}$.

## 5. Numerical discussion

**Figure 2.**
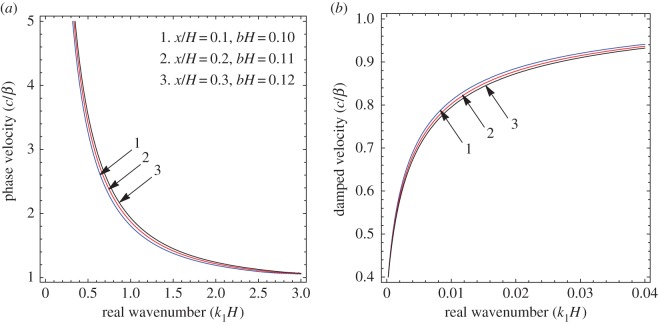
Case-I: Variation of (*a*) phase velocity and (*b*) damped velocity against real wavenumber for different values of x/H and *bH*.

**Figure 3.**
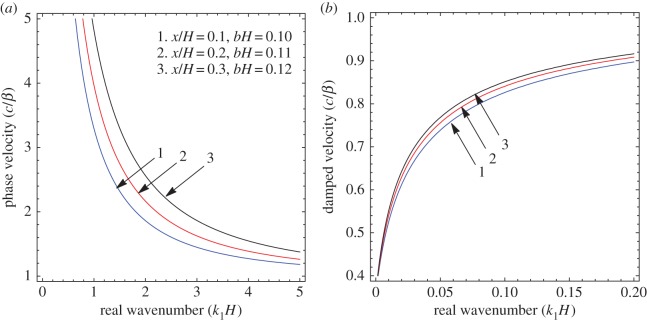
Case-II: Variation of (*a*) phase velocity and (*b*) damped velocity against real wavenumber for different values of x/H and *bH*.

**Figure 4.**
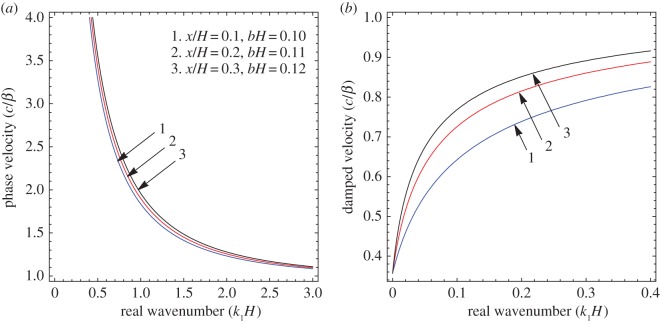
Case-III: Variation of (*a*) phase velocity and (*b*) damped velocity against real wavenumber for different values of x/H and *bH*.

**Figure 5.**
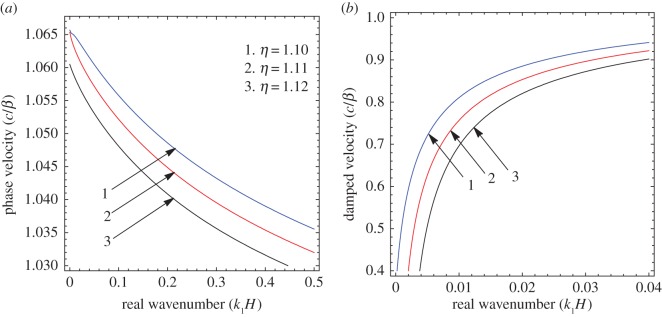
Case-I: Variation of (*a*) phase velocity and (*b*) damped velocity against real wavenumber for different values of sandiness parameter (*η*).

**Figure 6.**
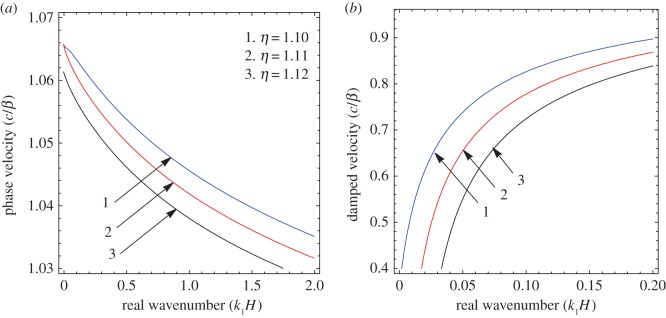
Case-II: Variation of (*a*) phase velocity and (*b*) damped velocity against real wavenumber for different values of sandiness parameter (*η*).

**Figure 7.**
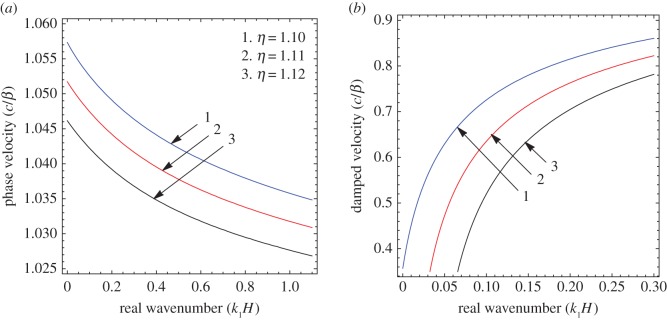
Case-III: Variation of (*a*) phase velocity and (*b*) damped velocity against real wavenumber for different values of sandiness parameter (η).

**Figure 8.**
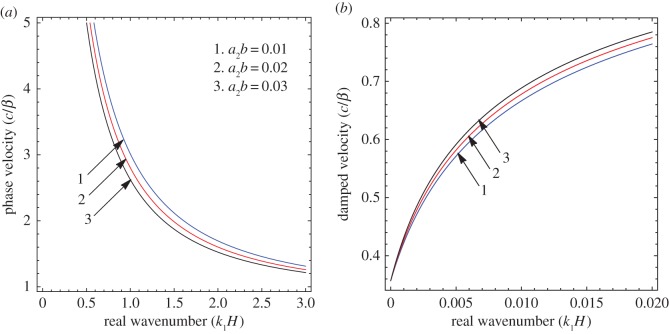
Case-I: Variation of (*a*) phase velocity and (*b*) damped velocity against real wavenumber for different values of corrugation parameter $\left(a_{2}b\right)$.

**Figure 9.**
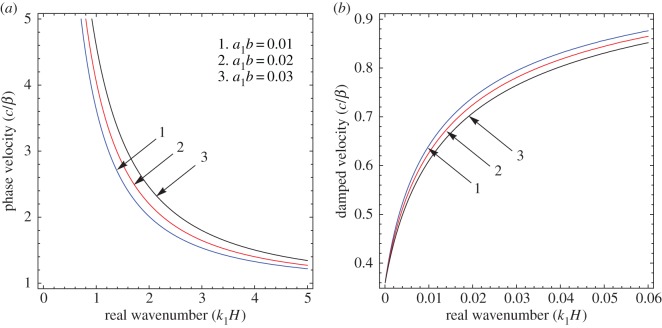
Case-II: Variation of (*a*) phase velocity and (*b*) damped velocity against real wavenumber for different values of corrugation parameter $\left(a_{1}b\right)$.

To illustrate the results of the dispersion and absorption characteristics, numerical calculations are presented using above-mentioned formulation with the help of Mathematica software. The set of parameter values has been considered from the references [25,26] (table 1). The illustrated results are presented graphically with three cases I, II and III. Figures 2, 5 and 8 have been plotted for case-I (i.e. $a_{1}=0$ and $a_{2}\neq 0$), figures 3, 6 and 9 for case-II (i.e. $a_{1}\neq 0$ and $a_{2}=0$) and figures 4 and 7 for case-III (i.e. $a_{1}\neq 0$ and $a_{2}\neq 0$).

**Table 1.** Properties of elastic materials.

medium	rigidity (N m${\;}^{-2}$)	density (kg m${\;}^{-3}$)
isotropic half-space [26]	$7.10\times 10^{10}$	3321
elastic medium with void pores [25]	$3.278\times 10^{10}$	1740
sandy half-space [26]	$7.84\times 10^{10}$	3535

**Figure 10.**
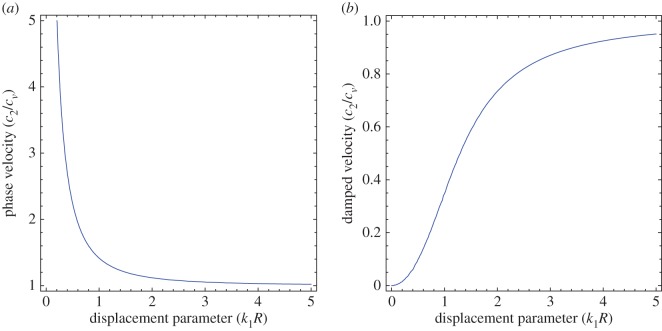
Variation of (*a*) phase velocity and (*b*) damped velocity against dimensionless displacement parameter of SH-waves of the second kind.

**Figure 11.**
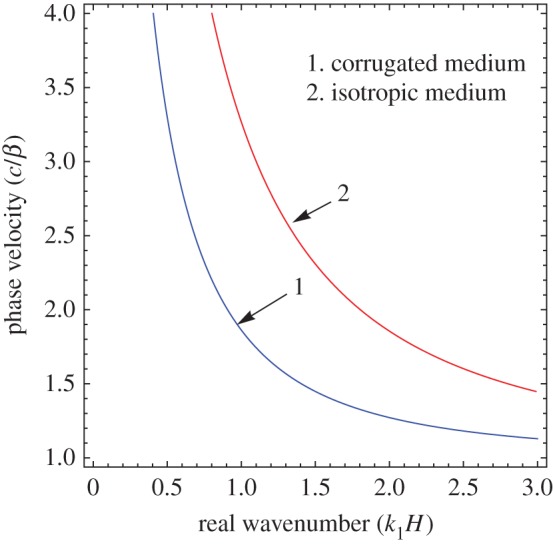
Comparison of phase velocity against real wavenumber in corrugated and isotropic medium.

Figures 2–9 represent the variation of dimensionless phase and damped velocity c/β against dimensionless real wavenumber $k_{1}H$ for the first kind of SH-waves. Each graph consists of two subfigures in which (*a*) corresponds to phase velocity and (*b*) to damped velocity. Figure 10 represents the dimensionless phase and damped velocity $c_{2}/c_{v}$ against dimensionless displacement parameter $k_{1}R$ for the second kind of SH-waves. In addition, a comparative study has been made to compare the velocity of SH-wave in classical case with respect to corrugated case in figure 11. In dispersion curves, we note that the phase velocity reduces with increase of wavenumber. In absorption curves, we observe that a maximum attenuation corresponding to the damped velocity increases initially and it becomes stable in higher range of wavenumber.

### 5.1. Effect of undulation and position parameters

Figures 2–4 show the variation of phase and damped velocity c/β with respect to the real wavenumber $k_{1}H$ for different values of undulatory parameter x/H and position parameter *bH*. The numerical values of x/H and *bH* for curves 1, 2 and 3 have been taken as (0.1, 0.10), (0.2, 0.11) and (0.3, 0.12), respectively. It is noticed that x/H and *bH* influence both the dispersion and absorption characteristics. In figure 2*a*, the nature of curves shift upwards for the increasing value of parameters x/H and *bH*, which concludes that the phase velocity of SH-wave increases. The effect of x/H and *bH* are significant in the low-frequency range, while the curves get accumulated for large values of frequency, which means these parameters have much less effect for higher wavenumber. On the other hand, it is clear from figure 2*b* that x/H and *bH* have negligible effect on the damped velocity for low frequency in comparison with the high-frequency range. The damped velocity decreases with increasing value of x/H and *bH*. Hence the phase and damped velocity have opposite nature with increasing value of undulatory and position parameter in the absence of upper corrugated boundary.

In figure 3*a*, the curves are very much influenced by the parameters x/H and *bH* over the considered frequency range. Figure 3*b* illustrates the effect of x/H and *bH* on damped velocity is much larger for high frequency in comparison with the low-frequency range. It is noted that the phase and damped velocity of SH-waves increase with increasing value of x/H and *bH* in the absence of lower corrugated boundary.

Figure 4*a* depicts the effect of parameters x/H and *bH* on dispersion characteristics of SH-wave, which establishes the phase velocity increases with the increase of parameters x/H and *bH*. Figure 4*b* shows the parameters x/H and *bH* affect the absorption characteristics significantly. It is observed that x/H and *bH* have positive effect on the phase and damped velocity of SH-waves in presence of corrugated boundary interfaces.

### 5.2. Effect of sandiness parameter

The figures 5–7 are associated with the variation of sandiness parameter *η* on the phase and damped velocity with respect to real wavenumber. To study the effect, the numerical values of sandiness parameter *η* have been considered as 1.10, 1.11 and 1.12 for cases I, II and III, respectively. We observe that *η* has a very significant effect on the dispersion and absorption characteristics of SH-waves throughout the considered frequency range in all cases. Figures 5*a*–7*a* illustrate, as the value of *η* increases the dispersion curve shifts downwards; this trend irradiates that the phase velocity decreases with the increase of sandiness parameter *η*. Figures 5*b*–7*b* show the damping nature of SH-waves for different values of *η*. As the value of *η* increases, the damped velocity decreases. Hence it can be concluded that the increasing value of *η* disfavours the dispersion and absorption characteristics of SH-waves in all cases.

### 5.3. Effect of corrugation parameters

The effect of corrugation parameters $a_{2}b$ and $a_{1}b$ on the SH-waves are exhibited in figures 8 and 9, respectively. Figure 8*a*,*b* shows the variation of phase and damped velocity for different values of $a_{2}b$ in the absence of upper corrugated boundary surface. The numerical values of $a_{2}b$ for curves 1, 2 and 3 have been considered as 0.1, 0.2 and 0.3, respectively. It can be observed that there is a significant effect of $a_{2}b$ on the phase velocity of SH-waves. It holds a very negligible effect on the low-frequency compared with the high-frequency regime. In addition, the phase velocity decreases with the increase of $a_{2}b$, whereas damped velocity increases. Figure 9*a*,*b* exhibits the variation of phase and damped velocity for different values of $a_{1}b$ in the absence of lower corrugated boundary surface. The values of $a_{1}b$ for curves 1, 2 and 3 have been considered as 0.01, 0.02 and 0.03, respectively. It is noted that the corrugation parameter $a_{1}b$ has a cogent effect on the phase velocity of SH-waves over the considered frequency range. Moreover, there is a positive relationship between $a_{1}b$ and phase velocity, i.e. phase velocity increases with increase of $a_{1}b$, whereas the damped velocity has an inverse relationship with $a_{1}b$.

The second kind of SH-wave velocities are exhibited in figure 10. The phase and damped velocity $c_{2}/c_{v}$ have been plotted from equations (3.18) and (3.19) with respect to displacement parameter $k_{1}R$ by taking a specified value of *t* and dimensionless parameter *κ*. Figure 10*a*,*b* illustrates that the phase velocity decreases with the increase of $k_{1}R$, whereas damped velocity increases.

This is evident from the comparative study as shown in figure 11, the dispersion curve associated with the isotropic medium is lying above with respect to corrugated medium. Hence this fact suggests that the SH-waves propagate even faster in isotropic medium than a layered corrugated structure.

## 6. Conclusion

The propagation of SH-waves in elastic medium with void pores between two half-spaces is studied in detail. The dispersion and absorption equations for SH-waves are derived separately in closed forms. The sandiness parameter, undulatory parameter, position parameter and corrugation have significant effects on dispersion and absorption characteristics (see the figures). Moreover, the following outcomes of the study can be highlighted:

— In elastic solids with void pores, more than one wavefront of SH-waves may exist, which satisfy the requirement for surface waves, i.e. to decay with the depth, whereas in the elastic case there is only ever one wavefront. Both wavefronts of SH-wave are dispersive in nature.
— The phase velocity of SH-waves decreases with increase in wavenumber.
— The damped velocity of SH-waves increases gradually with wavenumber and thereafter attenuates in high range of wavenumber.
— With the increase of sandiness parameter, the dispersion and absorption curves shift downwards. This trend shows that the phase and damped velocities of SH-waves decrease with the increase of sandiness parameter. It is observed that the sandiness parameter has a cogent effect on dispersion and absorption curves.
— The undulatory parameter and position parameter have harmonious effect on the phase velocity while it has inharmonious effect on the damped velocity. The undulatory parameter, position parameter and corrugation have a slight effect on damped velocity in the low range of wavenumber and significant effects in the higher range of wavenumber.
— As a particular case, the dispersion relation for the propagation of SH-waves at the common elastic medium between two different homogeneous isotropic half-spaces is validated with the results [24]. It is also noted that the damping part associated with dispersion relation in propagating the waves in an elastic medium sandwiched between two dissimilar isotropic half-spaces vanishes.

## Data accessibility

The datasets used in this work are described in the manuscript which is also cited as [24,25].

## Appendix A

Γ1=Re[k](H−f1(x)+f2(x))c2β2−1Γ2=δ(H−f1(x)+f2(x))c2β2−1; δ=Im[k]Re[k]=k2k1T1=c2β2−1(ημ0μ11−c2β02+μ2μ11−c2β22)T2=c2β2−1[ηf 1′(x)(1−μ0μ1)−f 2′(x)(η−μ2μ1)]T3=η(c2β2−1)−μ0μ1μ2μ11−c2β021−c2β22−f 1′(x)f 2′(x)(η−μ2μ1)(1−μ0μ1)T4=f 1′(x)μ2μ1(1−μ0μ1)1−c2β22+f 2′(x)μ0μ1(η−μ2μ1)1−c2β02Γ11=Re[k](H+a2cos⁡(bx))c2β2−1Γ21=δ(H+a2bsin⁡(bx))c2β2−1T11=T1T21=a2bsin⁡(bx)(η−μ2μ1)c2β2−1T31=η(c2β2−1)−μ0μ1μ2μ11−c2β021−c2β22T41=−a2bsin⁡(bx)μ0μ1(η−μ2μ1)1−c2β02Γ12=Re[k](H−a1cos⁡(bx))c2β2−1Γ22=δ(H−a1cos⁡(bx))c2β2−1T12=T1T22=−a1bsin⁡(bx)η(1−μ0μ1)c2β2−1T32=T31T42=−a1bsin⁡(bx)μ2μ1(1−μ0μ1)1−c2β02Γ13=Re[k][H+(a2−a1)cos⁡(bx)]c2β2−1Γ23=δ[H+(a2−a1)cos⁡(bx)]c2β2−1T13=T1T23=bsin⁡(bx)[a2(η−μ2μ1)−a1η(1−μ0μ1)]c2β2−1T33=T31+(a1b)(a2b)sin2⁡(bx)(η−μ2μ1)(1−μ0μ1)T43=−bsin⁡(bx)[a1μ2μ1(1−μ0μ1)1−c2β22+a2μ0μ1(η−μ2μ1)1−c2β02].
